# Fabrication and Characterization of the Porous Ti_4_O_7_ Reactive Electrochemical Membrane

**DOI:** 10.3389/fchem.2021.833024

**Published:** 2022-02-14

**Authors:** Guangfeng Qi, Xiaohui Wang, Jingang Zhao, Chunyan Song, Yanbo Zhang, Feizhou Ren, Nan Zhang

**Affiliations:** ^1^ Technical Test Center of Sinopec Shengli OilField, Dongying, China; ^2^ Testing and Evalution Research Co. Ltd. of Sinopec Shengli OilField, Dongying, China

**Keywords:** Ti4O7, reactive electrochemical membrane, Ti, TiO_2_, thermal reduction

## Abstract

Preparation of the Magnéli Ti_4_O_7_ reactive electrochemical membrane (REM) with high purity is of great significance for its application in electrochemical advanced oxidation processes (EAOPs) for wastewater treatment. In this study, the Ti_4_O_7_ REM with high purity was synthesized by mechanical pressing of TiO_2_ powders followed by thermal reduction to Ti_4_O_7_ using the Ti powder as the reducing reagent, where the TiO_2_ monolith and Ti powder were separated from each other with the distance of about 5 cm in the vacuum furnace. When the temperature was elevated to 1333 K, the Magnéli phase Ti_4_O_7_ REM with the Ti_4_O_7_ content of 98.5% was obtained after thermal reduction for 4 h. Noticeably, the surface and interior of the obtained REM bulk sample has a homogeneous Ti_4_O_7_ content. Doping carbon black (0wt%-15wt%) could increase the porosity of the Ti_4_O_7_ REM (38–59%). Accordingly, the internal resistance of the electrode and electrolyte and the charge-transfer impedance increased slightly with the increasing carbon black content. The optimum electroactive surface area (1.1 m^2^) was obtained at a carbon black content of 5wt%, which increased by 1.3-fold in comparison with that without carbon black. The as-prepared Ti_4_O_7_ REMs show high oxygen evolution potential, approximately 2.7 V/SHE, indicating their appreciable electrocatalytic activity toward the production of •OH.

## 1 Introduction

Ceramic porous sub-stoichiometric Ti oxides (Ti_n_O_2n-1_, 4≤n ≤ 10), known as Magnéli phases, were first synthesized and characterized in the 1950s (Zhou et al., 2018). Among these oxides, Ti_4_O_7_ exhibits excellent performance owing to its unique structure, e.g., excellent corrosion resistance and outstanding electrical conductivity with a value of 1000 S cm^−1^, which is higher than the 727 S cm^−1^ of graphitized carbon (Walsh and Wills, 2010). In light of this, the Ti_4_O_7_ material has been widely used as a cathodic protection during the electrodeposition process, in batteries as either an electrode material or an additive to the active materials (Zhang et al., 2017). In addition, due to its stability under anodic polarization and high oxygen evolution overpotential, Ti_4_O_7_ has been recently utilized as the inert anode material for weekly sorbed •OH production in the field of electrochemical wastewater treatment, which shows comparable electrocatalytic activity to the boron-doped diamond (BDD) anode for pollutant degradation (Bejan et al., 2009).

Recently, depositing Ti_4_O_7_ films at the Ti surface has been manufactured successfully by the plasma coating approach, which is a widely spread technology for many types of applications (corrosion protection, abrasion resistance, thermal barriers, etc.) (Ganiyu et al., 2016; Oturan et al., 2017). This preparation process is mainly proceeded by two steps: 1) reduction of TiO_2_ with coke to produce Ti_x_O_2x-1_ powder and 2) plasma elaboration of Ti_x_O_2x-1_ on the Ti-alloy substrate at 10,000–15,000°C accompanied by conversion of all sub-oxides of Ti to Ti_4_O_7_ [46]. However, the application of this Ti_4_O_7_ electrode is greatly limited by mass diffusion since there is a thick stagnant boundary layer (∼100 μm) at the plate electrode surface (Liu and Vecitis, 2012; Zaky and Chaplin, 2013). The flow-through mode, which is defined as operating with convective flow perpendicular to the porous electrode surface, allowed for enhanced mass transport rates relative to the traditional parallel flow mode by restricting the diffusional distance of reactants to a length-scale on the order of the pore radius of the electrode (Vecitis et al., 2011; Zaky and Chaplin, 2013; Guo et al., 2016a; Guo et al., 2016b). Thus, significant attention has been recently paid to the integration of membrane filtration and electrochemical process, known as the Ti_4_O_7_ reactive electrochemical membrane (REM), for aqueous contaminant degradation because this operation mode promotes the reaction between the contaminant and •OH produced at the anode surface. As a result, REMs based on electrochemical advanced oxidation processes (EAOPs) are a cutting edge class of electrodes that hold great promise in revolutionizing water/wastewater treatment (Zaky and Chaplin, 2013).

As for the preparation of the Ti_4_O_7_ REM, TiO_2_ is typically used as the main feedstock due to its abundance and relatively low cost. The mechanical pressing of Ti_4_O_7_ powders, followed by thermal sintering, has been widely used to fabricate the Ti_4_O_7_ REMs. There are many conventional approaches available to synthesize the Ti_4_O_7_ materials under controlled reducing conditions at relatively high temperature using different reducers such as metals (Kitada et al., 2012), carbon (Ganiyu et al., 2016), carbonaceous organic materials (Guo et al., 2019), and reducing atmosphere (H_2_ and NH_3_) (Li et al., 2010; Zhang et al., 2013; Lin et al., 2018). However, most of the production methods are energy and time-consuming. For example, the Ti_4_O_7_ REM was synthesized by reduction of commercial TiO_2_ ultrafiltration membranes at 1333 K for 50 h in the H_2_ atmosphere (Guo et al., 2016b). As another example, an ultrafiltration membrane layer composed of Ti_4_O_7_ and Ti_6_O_11_ was obtained by dip-coating of a TiO_2_ layer on the inner surface of a tubular Al_2_O_3_ membrane, followed by a reduction step under 30% H_2_ in the Ar atmosphere at 1308K for 7 h (Geng and Chen, 2016). High processing temperatures over 1000°C and such long time not only inevitably leads to morphology deformation but also results in particle aggregation and dense low surface area materials, which limit its practical applications as electrode materials, where high surface area, a large number of active sites, and a porous structure are all important for the performance (Lin et al., 2018). In addition to the cases under the reducing atmosphere (H_2_ and NH_3_), the as-prepared Ti_4_O_7_ REMs did not have high purity since the residual reducing reagents and their derivatives eventually reduce the purity of the obtained Magnéli Ti_4_O_7_ REM. However, the gaseous reduction process usually brings about high costs and potential danger. The high preparation costs and rigorously controlled fabrication conditions (H_2_ atmosphere) might limit their commercial development to some extent. Overall, the control of the stoichiometry of Magnéli phases according to the choice of precursors and thermal treatment is an interesting challenge for material science.

In the present study, thermal reduction of TiO_2_ by metal Ti under the vacuum condition was developed to prepare the Ti_4_O_7_ REM with high purity. The influences of the preparation temperature and thermal sintering time were optimized for Ti_4_O_7_ REM preparation. The Ti_4_O_7_ REM was characterized using X-ray diffraction (XRD), scanning electron microscopy (SEM), and mercury intrusion porosimetry. The water filtration performance of these REMs was evaluated based on the pressure-normalized permeate flux. In addition, the electrochemical properties of the REMs were studied by sweep voltammetry (CV) and electrochemical impedance spectroscopy (EIS).

## 2 Materials and Methods

### 2.1 Reagents

TiO_2_ (99.99%, 25 nm particles) and Ti (99.9%, 10 nm particles) powder were purchased from Beijing Xingyuan Technology Co., Ltd. Carbon black (XC-72R) was bought from Cabot Corporation. Ethanol (EtOH) (99.99%, anhydrous), sodium sulfate (99.9%), potassium ferrocyanide (99.5%), and potassium ferricyanide (99.5%) were obtained from Sigma-Aldrich. All solutions were prepared using deionized water (18.2 MΩ cm^−1^ at 25°C).

### 2.2 Ti_4_O_7_ REM Fabrication

The Ti_4_O_7_ ceramic microfiltration membrane was prepared by the following route: TiO_2_ powder and carbon black were first mixed together and then put into a ZrO_2_ ball-milling bowl (500 ml in volume, Fritsch, Germany), where the load of carbon black was set to 7.5% of the mass of the TiO_2_ powder. A planetary ball-mill (Fritsch, Pulverisette 6, Germany) was utilized to mix the membrane contents, in which the grinding balls were a mixture of Nikkato ZrO_2_ balls in the diameters of 1, 2, 5, 10, and 20 mm. The volume of the balls and the material account for 1/3 of the volume of the ball mill tank, respectively. Anhydrous ethanol is used as a ball milling agent. After allowing it to dry at 60°C overnight, 10 g powders were mixed with 2–3wt% of ethanol and transferred to a disc mould and pressed under the pressure of 30 MPa by a hydraulic press (Specac, UK) to form the precursor of the REM. The membrane was first sintered in air for 4 h at 973 K for the removal of carbon black then was reduced in the presence of the Ti powder (about 4 g) in a vacuum (<8 × 10^–3^ MPa) tube furnace for the preparation of the Ti_4_O_7_ REM. The precursor of the Ti_4_O_7_ monolith and Ti powder were separated from each other, and the distance is about 5 cm. The heating/cooling rates were both 4 K min^−1^.

### 2.3 Physicochemical and Electrochemical Characterization

XRD analysis was performed to obtain the phase information of the Ti_4_O_7_ REM. The Scherrer equation was used to calculate the crystallite size. The Ti_4_O_7_ REM surface morphology was characterized by SEM. Mercury intrusion porosimetry was used to investigate the porous structures of the electrodes. The EIS and CV analyses were conducted with an electrochemical workstation (PGSTAT302N, Metrohm) and used a three-electrode setup with the Ti_4_O_7_ electrode as the working electrode, a platinum plate electrode (3 cm × 3 cm) as the counter electrode, and a saturated calomel electrode (SCE) as the reference electrode. EIS analysis was performed at the open circuit potential (OCP, 190 mV/SCE) over a frequency range of 10^−2^–10^4^ Hz in 100 mM Na_2_SO_4_ supporting electrolyte solution with the presence of 5 mM K_4_Fe(CN)_6_ and 5 mM K_3_Fe(CN)_6_ redox couple. CV measurements were recorded at a voltage step of 0.05 V in 100 mM NaClO_4_ electrolyte. The electrochemically active surface area was calculated based on CV analysis at a scan rate from 5 to 30 mV s^−1^ in a potential range of −0.2–0.5 V.

## 3 Results and Discussion

### 3.1 Ti_4_O_7_ REM Fabrication

**FIGURE 1:**
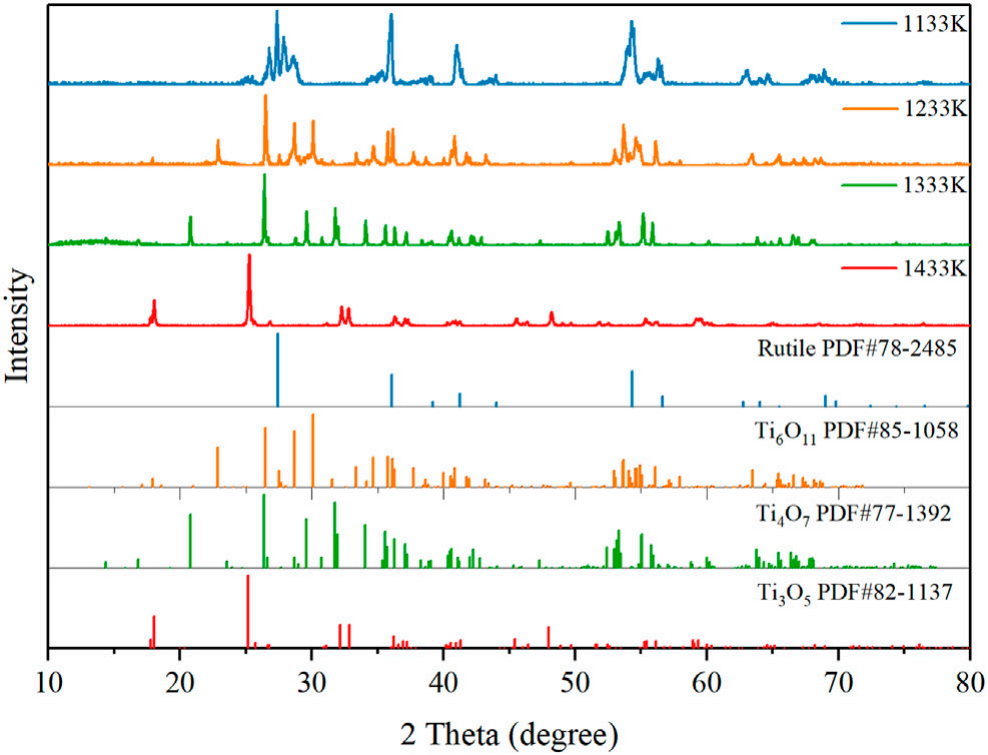
XRD patterns of the REM sample prepared at various temperatures.

The effect of thermal reduction temperature on the purity of the Ti_4_O_7_ REM was conducted in the range of 1133–1433 K. Figure 1 shows the XRD patterns of the REM samples prepared at various temperatures. It was observed that the color of the REM sample turned from white to light blue after thermal reduction at the reduction temperature of 1133 K. But the obtained REM sample was mainly rutile. This indicates that this reaction temperature was sufficient for the reduction of Ti^4+^ in anatase-TiO_2_ to Ti^3+^, while it was not high enough to transform anatase-TiO_2_ to the Magnéli phase. The phase transformation was observed at a sintering temperature of 1233 K, where the predominant phase was Ti_6_O_11_ and Ti_5_O_9_ was the marginal phase. When the temperature was elevated to 1333 K, the XRD patterns show the characteristic peaks for Ti_4_O_7_, and almost no peaks for other Magnéli phases suggests that a high purity Magnéli phase Ti_4_O_7_ REM was successfully fabricated. At this temperature, the Ti_4_O_7_ content of the samples was 98.5%, while the Ti_3_O_5_ content was only 1.5%. In contrast, when the reduction temperature was further increased to 1433 K, a single phase of Ti_3_O_5_ is formed, indicating that this reaction temperature was too high to synthesize pure Ti_4_O_7_.

**FIGURE 2:**
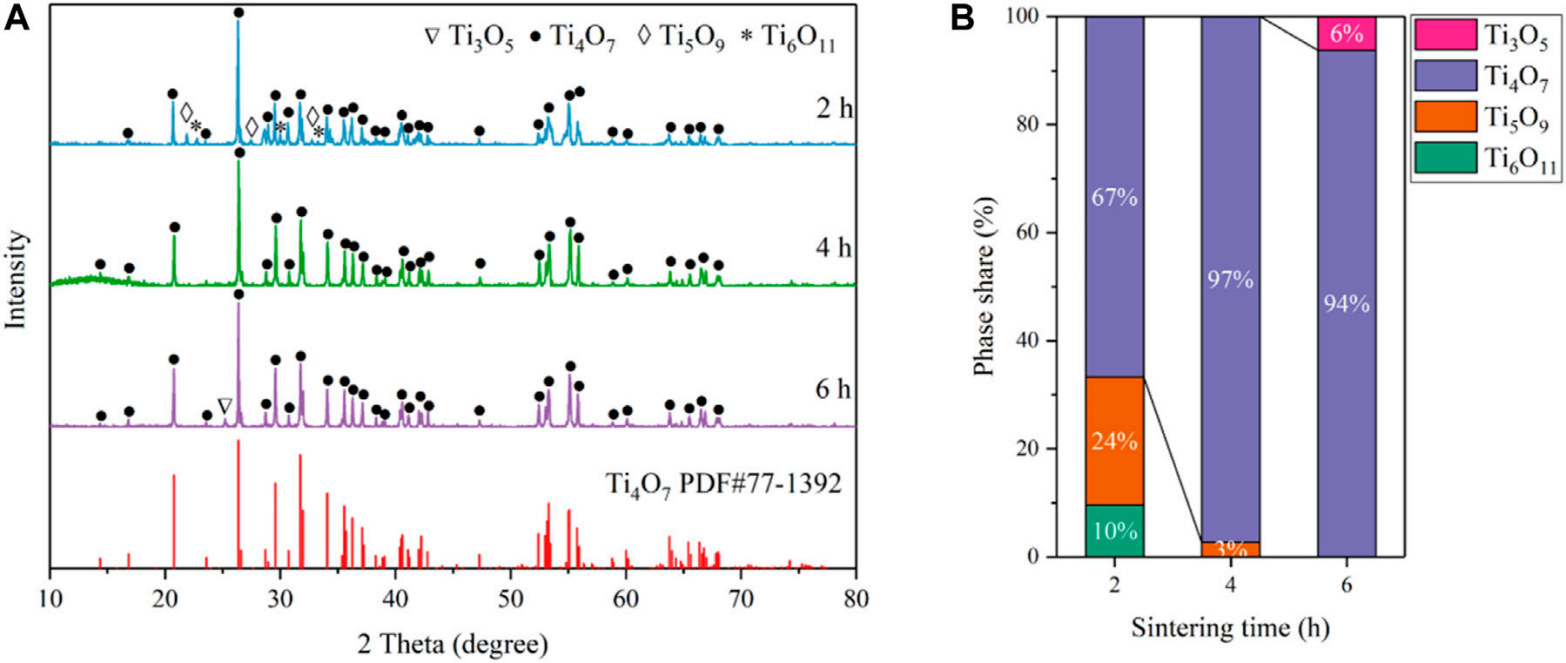
**(A)** XRD patterns of the as-prepared samples obtained at 1333 K for 2, 4, and 6 h; **(B)** Phase composition percentage.

To further find the optimal conditions for the synthesis of Ti_4_O_7_, the influence of thermal reduction time on the purity of the Ti_4_O_7_ REM was also studied. The XRD patterns of the as-prepared REM samples prepared at 1333K for 2, 4, and 6 h are shown in Figure 2. It is found that the Magnéli phase Ti_4_O_7_ was the major component at the thermal reduction time of 2–6 h. For 2 h, the as-prepared REM sample contains 24% Ti_5_O_9_, 10% Ti_6_O_11_, and 66% Ti_4_O_7_. When the thermal reduction time reached 4 h, XRD patterns show that Ti_5_O_9_ and Ti_6_O_11_ phases disappeared and Ti_4_O_7_ to be the main crystalline phase in the material with only <3% Ti_3_O_5_ left. Extending the reaction time to 6 h, the percentage of Ti_3_O_5_ phases increased slightly to 6%, and the percentage of Ti_4_O_7_ exhibited an insignificant change. These results mean that the thermal reduction time of 2 h is insufficient for the complete transformation of TiO_2_ to Ti_4_O_7_. However, as for 6 h, the increase of the miscellaneous peak, especially Ti_3_O_5_, indicates that the sample proceeded overreduction due to the longer thermal reduction time. These results illustrate the optimum reduction time for preparing the pure Ti_4_O_7_ REM was 4 h.

**FIGURE 3:**
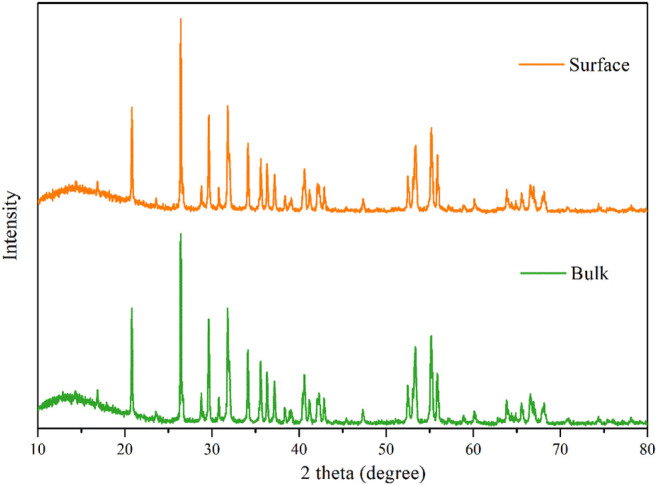
XRD patterns of the surface and interior of the REM monolith.

The XRD peaks for the reduced phases are relatively sharp (fwhm = 0.117° for 2 h, 0.084° for 4 h and 0.092° for 6 h) for the peaks at 26.4°, respectively. After accounting for instrumental broadening, the estimated crystallite sizes are 67 nm for 2 h, 90 nm for 4 h, and 119 nm for 6 h according to the Sherrer’s equation, while the crystallite size of the original TiO_2_ is 25 nm, indicating the crystallite growth. For further investigation of the phase composition of the productions, the XRD spectrums of the REM those were not grounded into powder are showed in Figure 3. In addition, the XRD peaks of the REM were perfectly coincident with those grounded into powder, meaning that the component of the REM bulk was homogeneous. It is noteworthy that as for the common carbothermal reduction approach with mixing carbon powder and TiO_2_, the surface and interior of the obtained bulk sample show different compositions (Tsumura et al., 2004; Toyoda et al., 2009).

### 3.2 Pore Properties Modification

**FIGURE 4:**
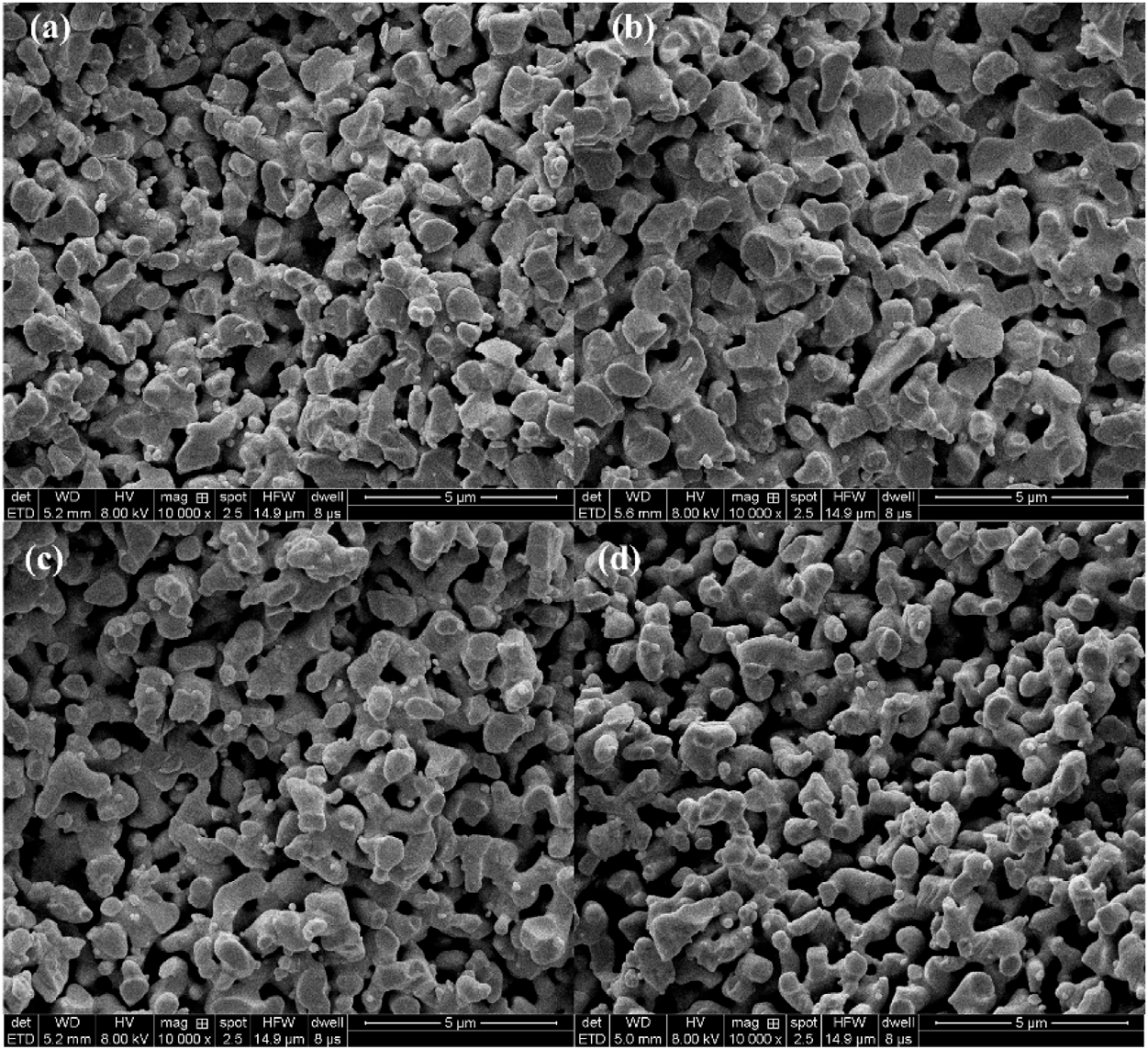
SEM image of the samples obtained at 1333 K with different carbon black contents of 0wt% **(A)** 1.5wt%, **(B)** 5wt%, and **(C)** 10wt% **(D)**.

**FIGURE 5:**
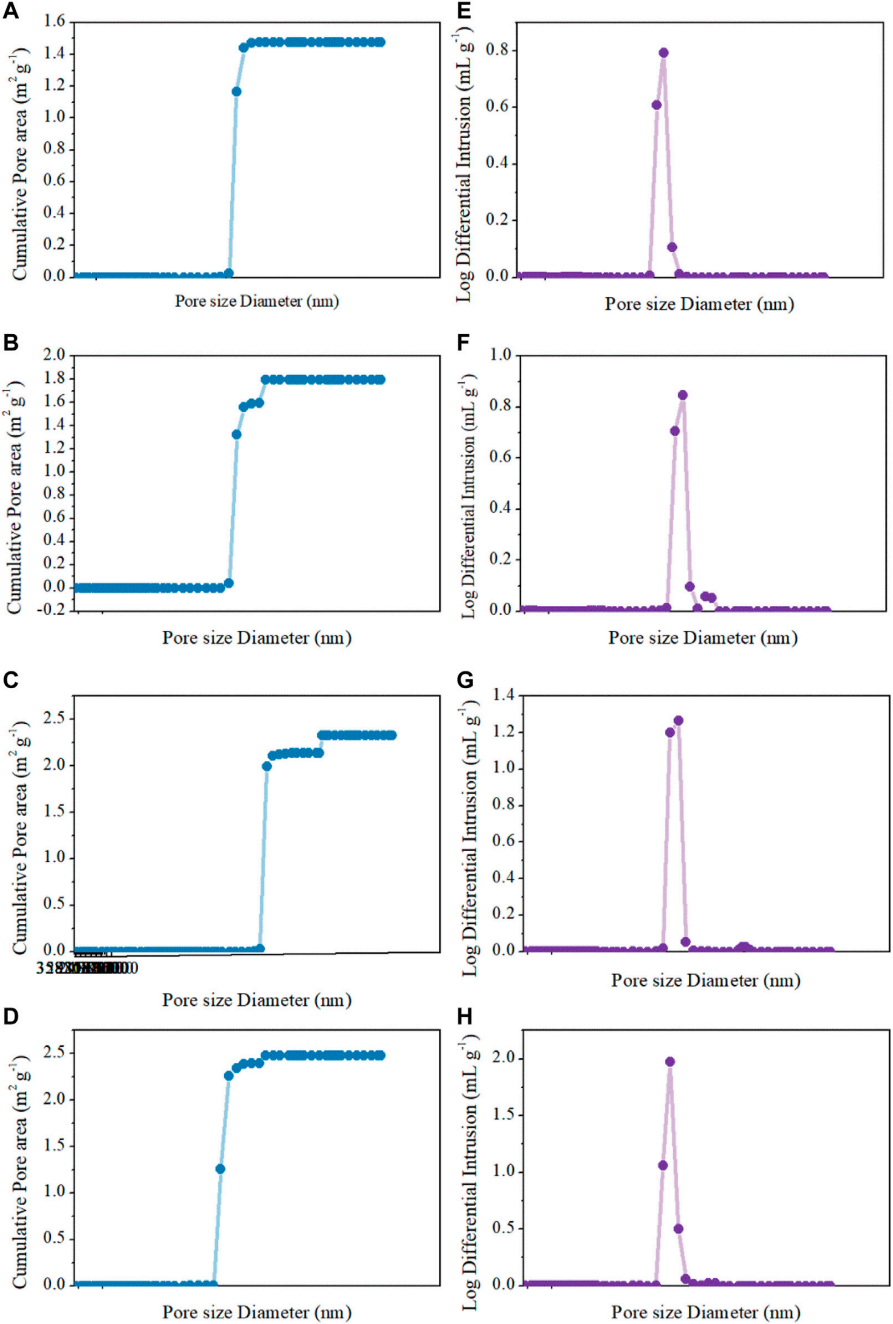
Cumulative pore area and log differential intrusion pore volume of Hg intrusion porosimetry analysis for **(A,E)** 0wt%, **(B,F)** 1.5wt%, **(C,G)** 5wt%, and **(D,H)** 15wt%.

**FIGURE 6:**
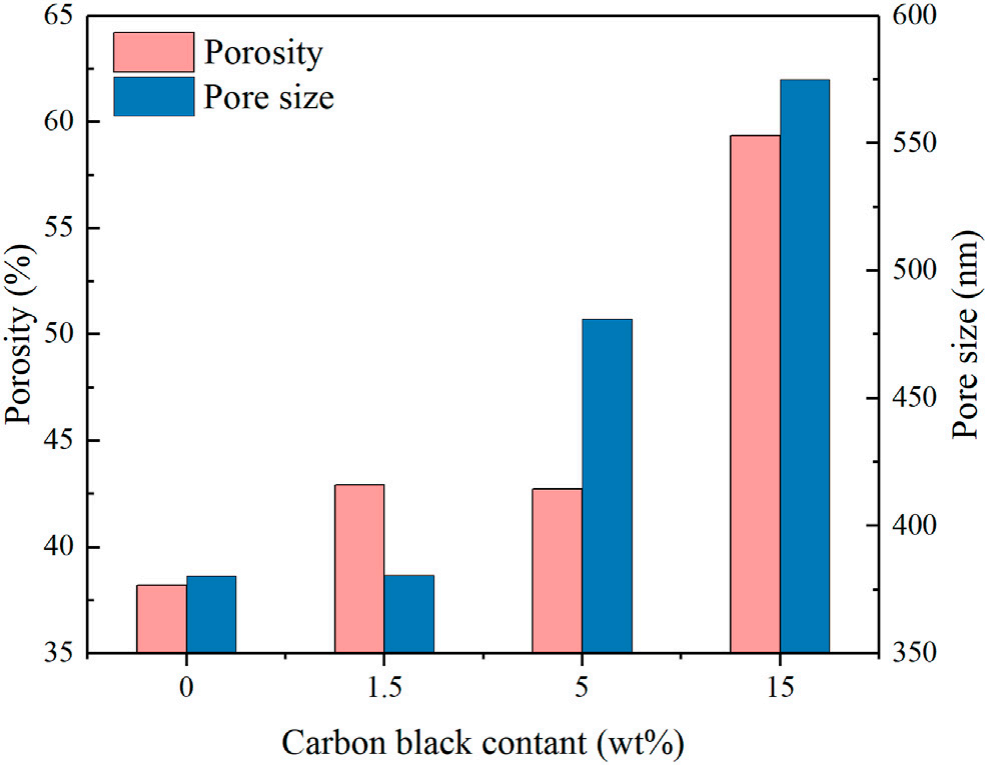
Porosity and pore size with different carbon black contents.

The morphology and pore structure of the REM were characterized by SEM Hg porsimetry, respectively. The SEM images in Figures 4A–D show the samples prepared at 1333 K with different carbon black contents of 0wt%, 1.5wt%, 5wt%, and 10wt%. The pore size of four samples was almost similar due to the fact that the grain-growth behavior mainly depends on the sintering time and temperature (Guan et al., 2016). The SEM images of the REM exhibit a three-dimensionally assembled structure with macro porosity, and the crystallites were indeed intimately fused or even consolidated to form large single-crystal particles on the micrometer scale. During the high-temperature reduction process, the agglomerates of solid particles and particle bonding resulted in the formation of the porous structure inside the cavity in the presence of the porosity-producing agent. Figure 4 shows that the pore size increased with the increasing carbon black content. The size of pores in Figure 4D seems to be larger than that in Figure 4A, which may be explained by the different carbon contents of these two samples. The detailed pore structure of these membranes was characterized by Hg porosimetry, and the results are presented in the Figure 5. It is demonstrated that the REM sample possessed uniform macropores of 0.4–0.5 μm (Figure 6). The almost same macropore diameter and volume indicate that the well-defined macroporous structure of the monolith was retained. These results are consistent with the results of SEM images. Moreover, the REM exhibits a pore size distribution with ∼99% of the measured surface area associated with pores <0.5 μm, and essentially, the entire pore volume attributed to pores with 0.2–0.5 μm diameters. As shown in Figure 5, based on the porosimetry results, the porous surface area for the membrane without carbon black was estimated as 1.477 m^2^ g^−1^ with the porosity(θ) and median pore diameter of 0.382 and 0.380 μm, respectively. As the content of carbon black was elevated to 1.5wt%, the porous surface area was enhanced to 1.797 m^2^ g^−1^ with the increase in the porosity by 4.7%, while no difference was observed in the pore size as compared with that without the addition of carbon black. The membrane with 5wt% carbon black has a porous surface area of 2.011 m^2^ g^−1^, median pore diameter of 0.481 μm, and its porosity is 0.427 which is the same with the former. The porosity for the REM with 15wt% carbon black was determined as 0.594, specific surface area of 2.477 m^2^ g^−1^, and median pore diameter of 0.575 μm. As the volume of carbon black increased, the change of pore size is slight, and the porosity has a gap of 21% between the samples without carbon black and with 15% carbon black. Micron-sized pores dominated the REM pore volume, which facilitates water transport through the REM at low applied pressures and is expected beneficial for facilitating interfacial mass transfer during the electrochemical reaction. Porosimetry analysis results show that the average pore diameter was closed to the median pore diameter (based on pore volume data) showing symmetrically distribution pore size. The addition of high carbon black content was not recommended since it would cause the crack of the REM monolith during the flow-through operation mode.

**FIGURE 7:**
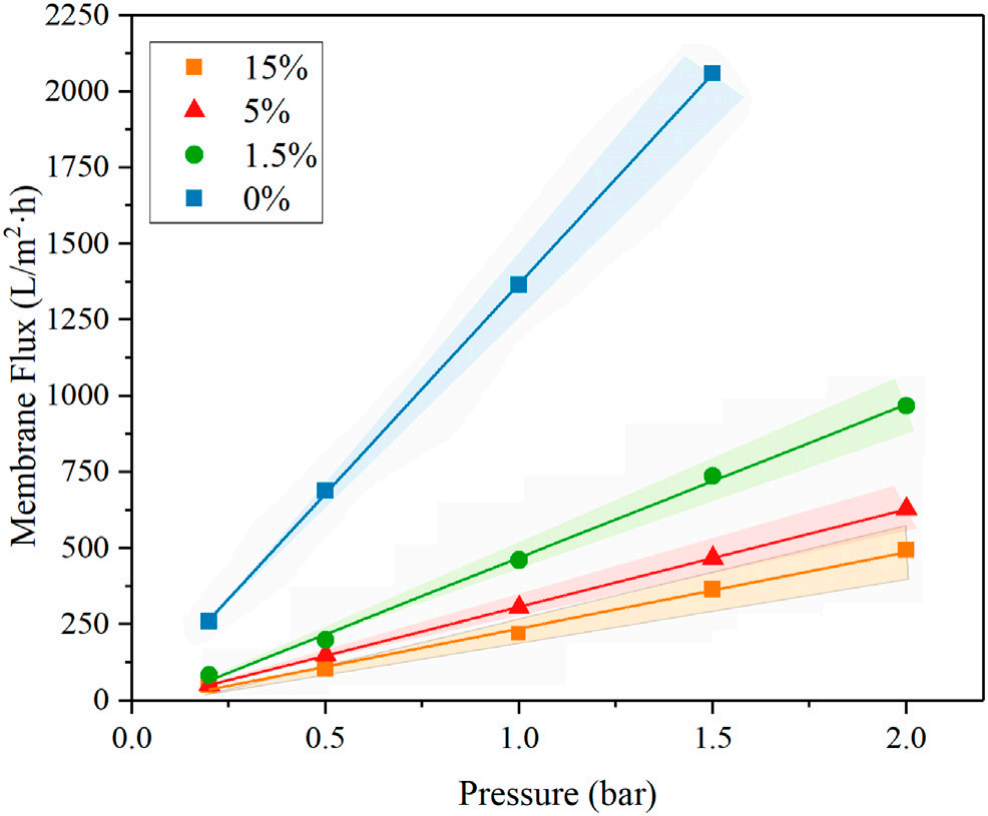
Pressure-normalized permeate fluxes of REMs with different carbon black contents.

A flow-through reactor was used to assess the permeation ability of REMs with ultra-pure water experiments which were carried out with the Ti_4_O_7_ REM. A digital gear pump was prepared to control the permeate flux and a pressure gage was used to record the hydraulic pressure. The evolution of the permeate flux with the carbon black content is plotted in Figure 7. It is found that the porosity and pore size both exerted the influence on the permeate flux. The REM without carbon black and with 1.5%wt carbon black have the same pore size of 380 nm but different porosity, e.g., 38 and 42%, respectively. The permeate flux of the latter increased 28% than the former. REMs with 1.5wt% and 5wt% carbon black have the same porosity of 0.42 and different pore size. With the bigger pore size and porosity, the increased extent of the permeate flux was much more evident. The permeate flux for the sample with the pore size of 480 nm increased by 57% than the sample with 380 nm pore size. The results of the REM membrane with 60% porosity and 580 nm pore size showed that the method improved the flux obviously with a 452% increase in the flux from 250 to 1379 L m^−2^ h^−1^ bar^−1^ than the original REM membrane. This indicates that a high content of carbon black was a crucial factor for the permeation ability of the REM membrane.

The resistance-in-series model was applied to evaluate the characteristics of the membrane flux; according to the Darcy law, the permeation flux (J) takes the following form:

$$J=\frac{\Delta P}{\eta R_{m}},$$ (1)

where J is the permeation flux (L/m^2^ h), ΔP (Pa) is the transmembrane pressure, η is the dynamic viscosity of the permeate, and $R_{m}$ (m^−1^) is the intrinsic membrane resistance. Table 1 made a summary of pressure-normalized permeate fluxes (LMH/bar) and intrinsic membrane resistance (10^10^/m) of the four samples. It can be seen that $R_{m}$ decreased with the increases of the carbon black content. $R_{m}$ of the original membrane is ∼6 times higher than that of the membrane with 15 wt% content of carbon black, which suggest that a lower membrane resistance can be achieved by simply increasing the content of carbon black.

**TABLE 1:** The effect of carbon black content on the property of REM samples.

Carbon black contents (wt%)	0	1.5	5	10
Pressure-normalized permeate fluxes (LMH/bar)	250 ± 10	320 ± 1	503 ± 12	1379 ± 8
Intrinsic membrane resistance (10^10^/m)	9.25	7.3	4.83	1.59
Determinate coefficient *R* ^2^	0.9869	0.9946	0.9979	0.9997

### 3.3 Electrochemical Characterization

**FIGURE 8:**
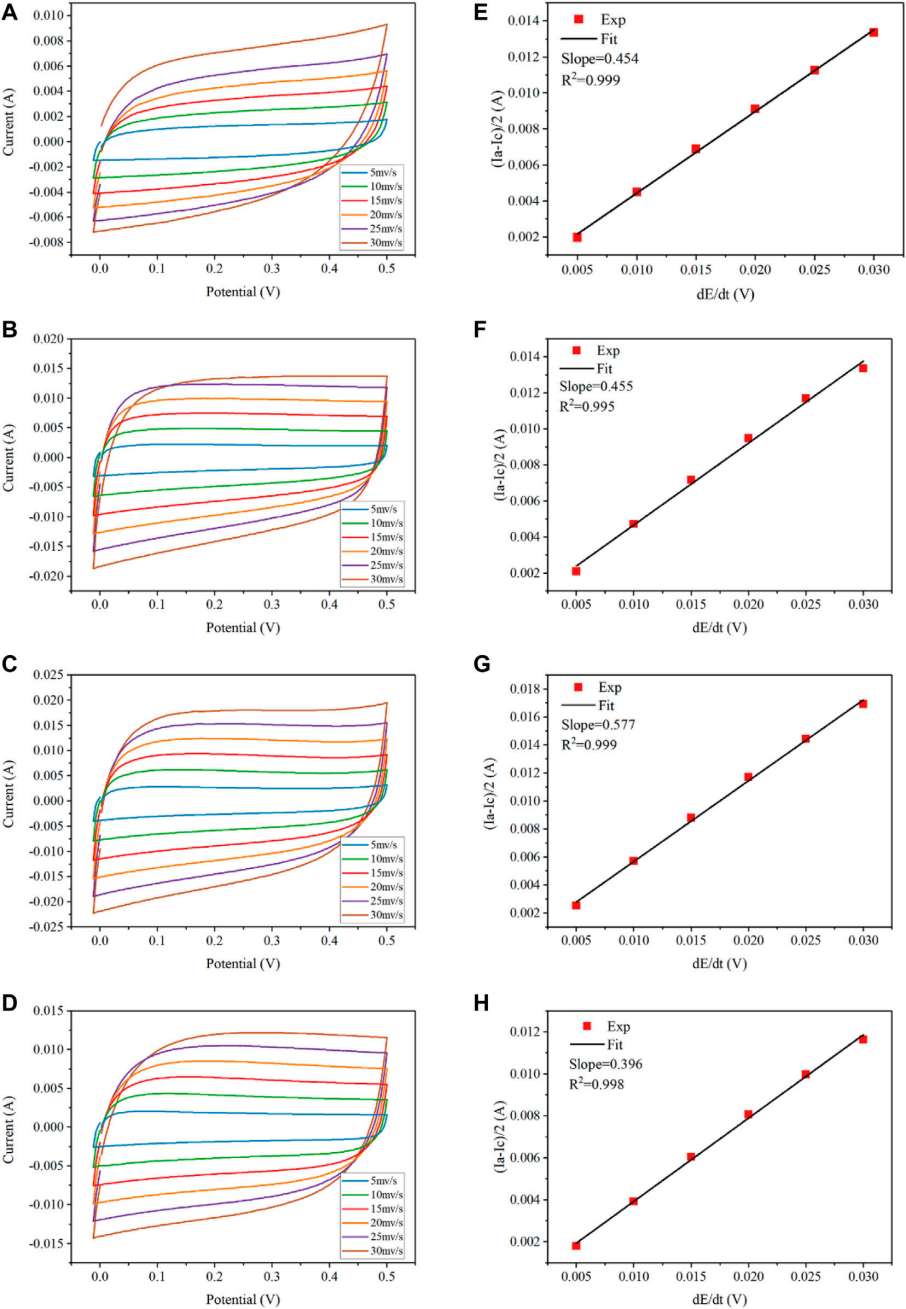
Determination of double-layer capacitance at different scan rates (5–30 mV s^−1^) for REMs using CV in the 100 mM Na_2_SO_4_ electrolyte solution. CV scans: **(A)** 0wt%, **(B)** 1.5wt%, **(C)** 5wt%, and **(D)** 15wt%. Corresponding plots of charging currents versus scan rates: **(E)** 0wt%, **(F)** 1.5wt%, **(G)** 5wt%, and **(H)** 15wt%.

The measurements of the electrochemically active surface area of each electrode were conducted within the potential region between hydrogen and oxygen evolution reaction, i.e., −0.2–0.5 V/SCE, at the sweep rates of 5–30 mV/s. As shown in Figure 8, the capacitive current decreases linearly with lowering sweep rates so that the apparent capacitance can be calculated from the slope of the charging current *vs* sweep rate. The double layer capacitances $\left(C_{dl}\right)$ were determined based on the CV test according to Eq. 2.

$$\frac{I_{a}-I_{c}}{2}=C_{dl}\mathrm{v,}$$ (2)

where $I_{a}$ and $I_{c}$ represent the measured plateau currents at 0.25 V/SCE, and v is the scan rate (V/s). The surface roughness factor and electroactive surface area were calculated according to the previous study with the use of the electrode geometric surface area. Assuming a double layer capacitance of 60 μF/cm^2^ for a surface of oxides, the roughness factors “r” can be estimated as follows:

$$\mathrm{r=}\frac{C_{\mathrm{dl}}}{C_{0}}.$$ (3)

Comparative cyclic voltammograms of the four REM membranes with a nominal geometric surface area of 3.38 cm^2^ in the potential range of −0.2–0.5 V/SCE are shown in Figure 8. It was calculated that the REM samples without doping carbon black and with 1.5% carbon black have the almost same average roughness (2557–2563). As for 5wt% carbon black, the calculated surface roughness was 3250 and the total electroactive surface area was 1.1 m^2^, which accounted for approximately 54.6% of the porous surface area measured by Hg porosimetry and increased by 1.3-fold in comparison with that without carbon black. However, the REM membrane obtained with doping 15wt% carbon black has the lowest surface roughness at 1953. In summary, the electrochemically active area (i.e., the roughness factor) of the porous Ti_4_O_7_ electrode is 3 orders of magnitude higher than the apparent surface area of the REM membrane, which was favorable for the electrochemical reaction.

**FIGURE 9:**
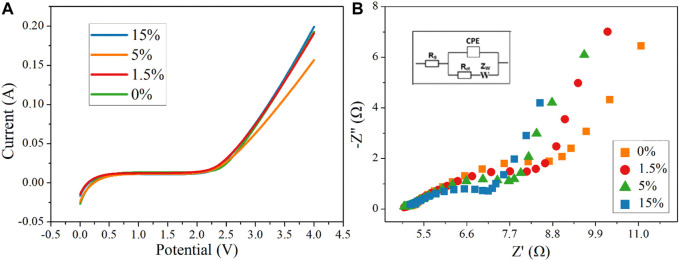
LSV **(A)** and EIS **(B)** curves of REMs.

A stepwise increase in potential was implemented by recording the current response to obtain the water oxidation potential in the Na_2_SO_4_ electrolyte (Figure 9). For Ti_4_O_7_, there was a negligible response of the current when the potential was below 2.7 V/SHE. Further increasing potential resulted in an apparent enhancement of the current, indicating the potential for water oxidation was within the range of 2.5–2.7 V/SHE. This result was higher than that of the oxygen evolution potential of 2.5 V/SHE reported by Smith et al. and reached the highest reported value of 2.7 V/SHE (Miller-Folk et al., 1989; Graves et al., 1991; Kolbrecka and Przyluski, 1994; Grimm et al., 1998). Thus, we can infer that the as-prepared Ti_4_O_7_ REM exhibits an appreciable electrocatalytic activity toward the production of •OH.

Figure 9B shows the EIS curves of REMs at room temperature. Each of the curves consists of a straight line (at low frequency) and a depressed semicircle (at high frequency), which are related to the ion diffusion in the bulk of the electrode and the charge transfer process at the electrolyte–electrode interface, respectively. The Rs displays the internal resistance of the electrode and electrolyte, and the charge-transfer impedance (R_ct_) is expressed vividly by the depressed semicircle in the intermediate frequency region. As shown in Figure 9B and Table 1, Rs values of Ti_4_O_7_ were estimated to be 17.1 ± 0.17, 17.53 ± 0.18, 18.8 ± 0.19, and 19.6 ± 0.20 Ω, respectively, indicating a minimal difference between the conductivity of these REMs. But it can be seen that as the content of carbon black increased, the value of R_s_ increased, which may be associated with the porosity of the REM membrane samples. The existence of a large number of voids reduced the effective cross-section of current conduction and therefore decreased the conductivity. Notably, the charge transfer resistances (R_ct_) of these REMs were 3.57 ± 0.36, 3.68 ± 0.37, 4.14 ± 0.41, and 5.19 ± 0.52 Ω, respectively, which were three orders of magnitude smaller than that for the graphite plate electrode (You et al., 2016; Xie et al., 2020), suggesting a much higher charge transfer capacity of Ti_4_O_7_.

### 3.4 Thermal Reduction Mechanism

In this study, the Ti_4_O_7_ REM fabrication process combined the following two steps: 1) the oxygen atoms in the TiO_2_ monolith reunited to oxygen and then effused into the vacuum environment at the higher reaction temperature and 2) the emanated oxygen is captured by the Ti powder. Thermodynamically, the reaction driving force enables titanium oxidation and TiO_2_ reduction. For step one, the general reaction can be given as

$$2\left(n-1\right)Ti_{n}O_{2n-1\left(s\right)}=2ni_{n-1}1O_{2n-3\left(s\right)}+O_{2\left(g\right)},$$ (4)

where Ti_n–1_O_2n–3_ and Ti_n_O_2n–1_ are the closest compounds in the Ti–O binary phase diagram. In these reactions, only oxygen is the gas phase, and others are pure solid. Then, the Gibbs free energy could be written as

$$\Delta_{r}G=\Delta_{r}G^{\theta }+\frac{1}{2}\mathrm{RTln}\frac{p_{O_{2}}}{P^{\theta }},$$ (5)

where $p_{O_{2}}$ is the partial pressure of O_2_, $p^{\theta }$ is the standard atmospheric pressure, and R is the universal gas constant. Due to the experiment being conducted in a vacuum, the practical pressure of oxygen is smaller than the total pressure of the vacuum chamber and certainly standard atmospheric pressure. This makes the value of $\mathrm{RT}\ln\frac{p_{O_{2}}}{p^{\theta }}$ negative, i.e., the Gibbs free energy change of the reaction at T temperature is minor as compared with the standard Gibbs free energy change, making decomposition of the metal oxide easier to occur. On the other side, the equation shows that the decomposition temperatures of the metal oxide depend on the oxygen pressure. Thus, the vacuum degree was also the critical factor for the successful fabrication of Ti_4_O_7_ REM samples.

When $\Delta_{r}G$ = 0, i.e., at the equilibrium, the relationship between temperature T and $\ln\frac{p_{O_{2}}}{p^{\theta }}$ was obtained:

$$\;\Delta_{r}G^{\theta }=-\frac{1}{2}\mathrm{RT}\ln\frac{p_{O_{2}}}{p^{\theta }}.$$ (6)

**FIGURE 10:**
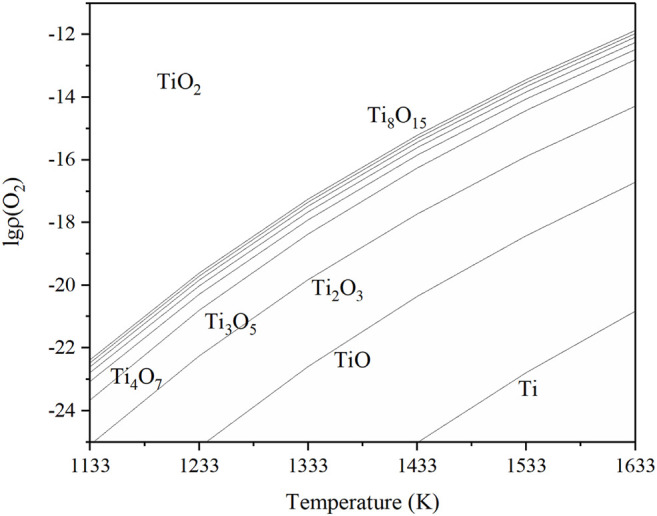
Phase stable diagram of the Ti–O system.

Based on the data of the Ti–O system, the diagram of this relationship is shown in Figure 10, called the Ti–O system phase stable diagram.

From Figure 10, Ti_4_O_7_ could be synthesized from TiO_2_ if the oxygen partial pressure and temperature were accurately controlled. If the system oxygen partial pressure is higher than the equilibrium value, the metal will be oxidized, and if it is lower than the equilibrium value, then the oxide will be reduced. The lower the temperature was, the wider the range of the oxygen partial pressure for Ti_4_O_7_ existed. When the temperature was adjacent to 1333 K and $\mathrm{lg}\frac{p_{O_{2}}}{p^{\theta }}$ < −17.9, the domain of Ti_4_O_7_ was expanded.

Table 2 is the equilibrium oxygen partial pressure of reactions in the Ti-O system at the temperature of 1233, 1333, and 1433 K. As the temperature increases by every 100 K, the oxygen partial pressure can increase by several orders of magnitude. It means that raising the temperature could make the reaction occur more easily and more distinguished; thus, the possibility to fabricate a single-phase material is greatly raised. However, it should be noted that the treatment time must be reduced at a high thermal reduction temperature since it probably induced the overreduction of TiO_2_ to some Ti_n_O_2n-1_, such as Ti_3_O_5_ (Figure 2).

**TABLE 2:** Equilibrium oxygen partial pressure of reactions in the Ti–O system at a specified temperature.

Reactions	Sintering temperature T/K, when $\Delta_{r}G^{\theta }=0$	$\mathrm{lg}\frac{p_{O_{2}}}{p^{\theta }}$
12TiO_2_ (s) =2Ti_6_O_11_ (s)+O_2_ (g)	1233	−19.8
8TiO_2_ (s) =2Ti_4_O_7_ (s)+O_2_ (g)	1333	−17.9
6TiO_2_ (s) =2Ti_3_O_5_ (s)+O_2_ (g)	1433	−16.3

**FIGURE 11:**
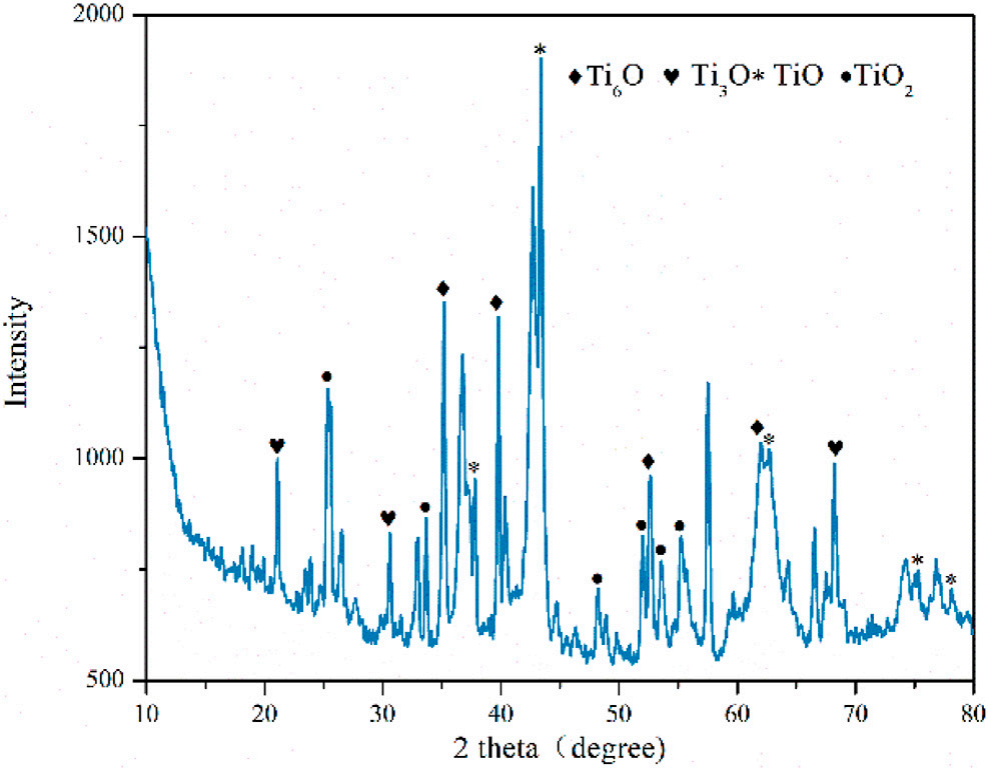
XRD patterns of the oxidation products of the Ti reducing reagent.

Excess amount Ti was use to decrease the oxygen pressure and ensure more complete reduction of TiO_2_ during the process. It has been shown that the growth of titanium oxide mainly in the form of rutile at and above 10^−7^ MPa and at the temperature below 1573 K. However, at lower pressures and at higher temperatures and after oxygen saturation of the a-phase, all the oxides of titanium (e.g., Ti_2_O, TiO, Ti_2_O_3_, Ti_3_O_5_, and TiO_2_) are formed as reaction products depending on the oxygen pressure and elapsed time of reaction. As a matter of fact, the last oxidized product is not TiO_2_ but the mixture of various titanium oxide (Figure 11). As the temperature was 1273 K, the oxygen partial pressure $p_{O_{2}}=10^{-18.6}$ MPa which is much lower than 10^−7^ MPa. The Ti powder used in our experiments is the nano-scale powder, which created a large surface area to contact with oxygen to increase the dissociation pressure of TiO_2_ as much as possible kinetically.

## 4 Conclusion

In this study, the high purity Ti_4_O_7_ REM was successfully synthesized by mechanical pressing of TiO_2_ powders followed by thermal reduction to Ti_4_O_7_ using the Ti powder as the reducing reagent. Carbon black was introduced to the monolithic TiO_2_ precursor to control the pore size and morphology of the Ti_4_O_7_ REM. The pore properties modification increased the electroactive surface area by approximately 1.3-fold, which increased the reactivity of the Ti_4_O_7_ REM toward outer sphere electron transfer reactions. The electrodes had high porosities (38–59%), which showed high permeate fluxes of up to 1379 ± 8 LMH bar^−1^. These results indicated that the Ti_4_O_7_ monolithic electrodes could find various electrochemical applications in water treatment and energy storage and conversion.

## Data Availability

The original contributions presented in the study are included in the article/Supplementary Material; further inquiries can be directed to the corresponding authors.
